# IL15N72D superagonist/IL15Rα-Fc fusion complex (ALT-803) exhibits anti-metastatic activity in murine breast tumor model

**DOI:** 10.1186/2051-1426-3-S2-P229

**Published:** 2015-11-04

**Authors:** Peter S Kim, Anna R Kwilas, Emily K Jeng, Hing C Wong, Jeffrey Schlom, James W Hodge

**Affiliations:** 1 National Cancer Institute, National Institutes of Health, Bethesda, MD, USA; 2 Altor BioScience Corporation, Miramar, FL, USA

## 

Interleukin (IL)-15N72D-superagonist-complexed with IL15Rα-Fc fusion protein (IL15-SA/IL15Rα-Fc, also known as ALT-803) has been reported to have greater anti-myeloma activity, due to its increased *in vivo* half-life and unique tissue biodistribution, than native IL-15 alone in murine models. In order to test the anti-tumor efficacy of IL15-SA/IL15Rα-Fc in a non-hematologic murine cancer model, we examined the monotherapy effect of IL15-SA/IL15Rα-Fc in non-tumor bearing mice and in the 4T1-breast tumor model. In non-tumor bearing Balb/c mice, IL15-SA/IL15Rα-Fc (1 µg i.p.), a 10-fold increase occurred in NK cells followed by CD8+ T cells (3-fold), both peaking on Day 3 post treatment. In examining NK cell population subsets, the greatest significant change was in high effector (CD11b+, CD27hi) NKs as compared with terminal effector (CD11b+, CD27lo) NKs on Day 3 post IL15-SA/IL15Rα-Fc-treatment, leading to increased NK function on a per-cell basis. CD8 subset analysis determined that IL15-SA/IL15Rα-Fc significantly increased IL15-responding, memory (CD122+, CD44+) CD8+ T cells, in particular those having the innate (NKG2D+, PD1-) phenotype. In 4T1 tumor bearing mice, IL15-SA/IL15Rα-Fc induced significant anti-metastatic activity, and thus consequently resulted in a longer median survival of IL15-SA/IL15Rα-Fc-treated mice following surgical resection of the primary tumor. Finally, T cell depletion revealed that the anti-metastatic property of IL15-SA/IL15Rα-Fc was dependent on CD8+ T cells and not CD4+ T cells. Altogether, these studies showed for the first time that IL15-SA/IL15Rα-Fc a) promoted the development of high effector NKs, b) enhanced per-cell function of NKs, and c) played a vital role in reducing tumor metastasis and ultimately survival.

